# The British Association and "Malaria"

**Published:** 1900-09-15

**Authors:** 


### The Hospital

A JOURNAL OF

The Medical Sciences and Hospital Administration.

VOL. XXVIII.—No. 729.] EDITED BY SIR HENRY BURDETT, K.C.B., AND SOLOMON C. SMITH, M.D., M.R.C.P. [SEPTEMBER 15, 1900.

### THE BRITISH ASSOCIATION AND "MALARIA."

THE meeting of the British Association for the Advancement of Science, under the presidency of so distinguished a member of the medical profession as Sir William Turner, afforded a specially suitable opportunity for bringing before its notice the latest researches made by Major Ross into the life history of the anopheles and its parasites, to which the human race is indebted for the intermittent fevers which have hitherto presented so formidable an obstacle to the colonisation of many countries by Europeans. We purposely avoid the use of the word "malaria" in this connection; because, now that the hypothesis which it expressed has been shown to be erroneous, the retention of the word can only serve to confer artificially prolonged vitality upon error. It may now be said to be indisputably proved that intermittent fever, of whatever variety, has one source and one alone—namely, the introduction into the blood of the human subject of parasites derived immediately from the salivary gland of a blood-sucking insect, and originally derived by the insect from the blood of an infected human subject. The word "mosquito" might with advantage follow the word "malaria," because the former is applied indiscriminately to many varieties of the gnat family, of which only one, the anopheles, has hitherto been found to convey the parasites, or to be an occasion of anything more than temporary discomfort from its bites. Hence it is that travellers, ignorant alike of medicine and of natural history, and rendered rash by their ignorance, have written to non-medical papers to dispute the conclusions established by the careful work of Major Ross and his fellow-labourers. Such writers say that they have been severely bitten by "mosquitoes" without contracting fever, or that they have contracted fever in a place where they never saw a "mosquito." The explanation of the former statement is either that the so-called "mosquitoes" were harmless culices, incapable, so far as is known, of becoming the hosts of the parasites, or else that the locality was one into which intermittent fever had not been introduced, and in which, therefore, the anopheles itself had never become infected. The explanation of the second is either that the bites of infected anopheles were inflicted during sleep, and failed to attract notice, or else that a certain time was required before the parasites had multiplied sufficiently to produce symptoms, and that, during this time, the traveller had passed away from the place where they were introduced into his body. As an illustration of the necessity for thus modifying accepted nomenclature, so as to avoid what South described as "the terrible imposture and force of words," we may mention that a book containing non-medical hypotheses about plague, just published by an author professedly scientific, after giving particulars of some experiments intended to show the rates of evaporation from, and of condensation in, the earth in different states of temperature, proceeds to say that "these experiments threw considerable light upon the influence of malaria, and explained some well-established facts known in reference to the influence of malaria, such as the circumstance that malarious countries can be traversed with impunity in the daytime but are very fatal at night." The well-meaning author has evidently been completely misled by the derivation of the word "malaria," and does not know that it only exists as a survival of an exploded error.

It may now be considered an established fact that countries in which intermittent fever prevails may be travelled in or lived in with perfect safety on the sole condition of the proper and effective employment of mosquito nets during the periods when the insects are active and feeding. This was practically known eighty years ago, and was stated in an article on "Malaria" which was published in the *Edinburgh Review* for 1822; but the explanation of the fact was necessary before its significance could be fully appreciated. It leaves untouched the far more important question of how far our knowledge of the cause of intermittent fever permits us to hope for its complete extinction. Nothing is known, of course, with regard to the primary origin of the parasite, but at present the anopheles seems to derive it only from man. To use the phrase of Koch, it is as easy to poison the parasite in human blood by the administration of quinine to the host as it would be to poison the host himself by the administration of arsenic; and he has therefore suggested that, given enough doctors and enough quinine, it would be possible to destroy every parasite in a given territory. To examine the blood of the whole native population, infantile and adult, of any country, and to administer quinine to all in whose blood the parasite was found, until it could be found no longer, would, however, be an undertaking before which the difficulties of enforcing vaccination would sink into insignificance; and it is therefore probable that the victory of civilisation over intermittent fever in the tropics will be only gradual, as it has been at home. People who aro still living can remember "ague" as a not uncommon malady in the fen districts of England. The haunts of the anopheles have been modified by drainage and cultivation, and the infected blood of the human sufferers has been freed from the parasites by quinine. The same order of events will ultimately be repeated elsewhere, and in the meanwhile those who have business in fever-stricken countries must follow the counsel of the Royal Society's Committee, must use mosquito nets effectively, and must avoid as far as possible both native dwellings and the clearings in which they stand.

